# Precision Oncology: Circulating Microvesicles as New Biomarkers in a Very Early Stage of Colorectal Cancer

**DOI:** 10.3390/cancers16101943

**Published:** 2024-05-20

**Authors:** Anastasios G. Kriebardis, Leonidas Chardalias, Christos Damaskos, Abraham Pouliakis, Nikolaos Garmpis, Sotirios P. Fortis, Aspasia Papailia, Christiana Sideri, Hara T. Georgatzakou, Effie G. Papageorgiou, Theodoros Pittaras, Gerasimos Tsourouflis, Marianna Politou, Ioannis Papaconstantinou, Dimitrios Dimitroulis, Serena Valsami

**Affiliations:** 1 Laboratory of Reliability and Quality Control in Laboratory Hematology (HemQcR), Department of Biomedical Sciences, School of Health & Caring Sciences, University of West Attica (UniWA), 12243 Egaleo, Greece; akrieb@uniwa.gr (A.G.K.); sfortis@uniwa.gr (S.P.F.); cgeorgatz@uniwa.gr (H.T.G.); efipapag@uniwa.gr (E.G.P.); 2 2nd Department of Surgery, Aretaieion University Hospital, Medical School, National and Kapodistrian University of Athens, 11528 Athens, Greece; lchardal@med.uoa.gr (L.C.); apapailia@uoa.gr (A.P.); johnpapacon@med.uoa.gr (I.P.); 3 Second Department of Propedeutic Surgery, Laiko General Hospital, Medical School, National and Kapodistrian University of Athens, 10679 Athens, Greece; kckont@med.uoa.gr (C.D.); ngarmpis@uoa.gr (N.G.); gtsourouflis@med.uoa.gr (G.T.); dimdimitr@med.uoa.gr (D.D.); 4 Second Department of Pathology, “Attikon” University Hospital, National and Kapodistrian University of Athens, 10679 Athens, Greece; apouliak@med.uoa.gr; 5 Hematology Laboratory-Blood Bank, Aretaieion Hospital, National and Kapodistrian University of Athens, 11528 Athens, Greece; christianakondylo.sideri@dcu.ie (C.S.); tpittaras@med.uoa.gr (T.P.); mpolitou@med.uoa.gr (M.P.)

**Keywords:** circulating microvesicles, microparticles, biomarkers, cancer, colorectal cancer, survival

## Abstract

**Simple Summary:**

Microvesicles (MVs) are essential for inter-cellular signaling in health and disease. We analyzed MV levels in colorectal cancer patients and assessed their release in early-stage colorectal cancer and survival. Considering that all types of MV were elevated beginning in the very early stages of the disease, we believe that the study of circulating MV levels could provide evidence for their use in the early detection of colon cancer in patients.

**Abstract:**

Background: The release of microvesicles (MVs) is an essential phenomenon for inter-cellular signaling in health and disease. The role of MVs in cancer is multidimensional and includes cancer cell survival, proliferation, and invasion. In this prospective study, we analyzed MV levels in colorectal cancer patients and assessed the importance of MV release in early-stage colorectal cancer and survival. Methods: This study included 98 patients and 15 controls. The characterization of MVs from human plasma was performed by flow cytometry using monoclonal antibodies. Results: The levels of total MVs and MUC-1-positive, tissue factor (TF)-positive, and endothelial cell-derived MVs (EMVs) were statistically significantly higher in the colon cancer patients than in the controls (*p* < 0.001). Furthermore, the subgroup of patients with very early-stage colorectal cancer also had statistically significant differences in the levels of the abovementioned MVs compared to the controls (*p* < 0.01). Highly differentiated tumors had lower levels of MUC-1-positive MVs (*p* < 0.02), EMVs (*p* < 0.002), and EMV/TF combinations (*p* < 0.001) versus those with tumors with low/intermediate differentiation. Conclusions: Our data demonstrate that the analysis of circulating MV levels in plasma could possibly become a tool for the early diagnosis of colon cancer at a very early stage of the disease.

## 1. Introduction

Microvesicles are a type of extracellular vesicle that include exosomes and apoptotic bodies [1]. Exosomes are formed by the inward budding of the plasma membrane, and their size is between 40 and 150 nm [2]. Apoptotic bodies arise from the fragmentation of apoptotic cells, and their size is greater than 1000 nm. MVs are between 150 and 1000 nm and are formed by the outward blebbing of the cell membrane [3]. Recent studies suggest a multidimensional role of microvesicles (MVs) in cancer cell survival, proliferation, and invasion [4,5]. MVs derived from tumor cells affect the surrounding microenvironment and distal organs through a variety of mechanisms including immune system inhibition, angiogenesis induction, oncogene transfer, and chemotherapy resistance [6,7]. 

MVs have garnered significant interest as potential biomarkers due to their ability to reflect the molecular profiles of their parent cells [8], including cancer cells [9], and their presence in various biological fluids, such as blood and urine [10]. Recent studies have shown promising results regarding the diagnostic and prognostic utility of microvesicles in colon cancer, with their cargo mirroring the aberrant signaling pathways and genetic alterations characteristic of the disease [11,12]. MVs can be easily obtained by liquid biopsies, namely blood samples, and provide more information about tumor characteristics, prognosis, metastatic potential and targetable pathways for treatment than a solid biopsy [13,14,15]. In addition, they can provide tumor-specific details such as the status of mutations, gene amplifications, and the transcriptome [16,17,18]. Moreover, advancements in isolation and characterization techniques, such as flow cytometry, nanoparticle tracking analysis, and mass spectrometry, have enhanced our ability to accurately detect and analyze microvesicles, further facilitating their clinical translation [19].

The prevention and treatment of colorectal cancer (CRC) is a global health challenge since CRC ranks as the second leading cause of cancer-related mortality [20]. Recent studies highlight the importance of microvesicles as biomarkers for the early detection of CRC [21], prognosis, and the prediction of response to therapy [22,23]. 

We performed a prospective study to assess the levels of different types of MVs in patients newly diagnosed with colorectal cancer and correlate them with disease and patient characteristics. As cancer treatment (surgery, chemotherapy, and radiotherapy) can induce the release of MVs from tumor and normal cells [24], all patients were included in our study prior to any treatment. 

## 2. Materials and Methods

### 2.1. Study Population

The present study included 98 patients diagnosed with CRC between March 2014 and December 2016 at participating centers. Blood samples were collected on the day before an elective surgery. Patients who received chemotherapy or radiotherapy before surgery and patients on anticoagulants and/or those with a history of thrombosis were not included in this study. A cancer-free control group matched for sex and age distribution was also evaluated. Demographic and medical history data were available for both groups. In addition, clinical and laboratory data, tumor location, staging, histological analysis data, outcome, and follow-up data were assessed. 

This study was conducted in accordance with the principles of the Declaration of Helsinki and approved by the Institutional Review Board of Aretaieio Hospital, the Institutional Review Board of Laiko Hospital, and the Research Ethics Committee, all belonging to the National and Kapodistrian University of Athens. Written informed consent was obtained from all participants (patients and controls).

### 2.2. Blood Sampling

Whole blood samples were collected from colon cancer patients and healthy subjects via an antecubital vein before surgery using a 21 gauge catheter (BD Vacutainer) and transferred in ethylenediaminetetraacetic acid (EDTA) and 3.2% sodium citrate blood collection tubes (BD Vacutainer Blood Collection Tubes, BD Biosciences, San Jose, CA, USA). 

### 2.3. Hematological and Screening Hemostasis Measurements

BC-3000 PLUS, MINDRAY Celltac E, MEK-7222 Κ, and NIHON KOHDEN automatic blood cell counters were used to obtain complete blood count through double measurements. 

### 2.4. Characterization of Cell-Derived Microvesicles from Human Plasma

Citrated blood samples were processed within 30 min of venipuncture using two successive centrifugations at 2.500× *g* spin for 15 min at 20 °C, as previously described [25]. The final platelet-free plasma supernatant was immediately frozen and stored at −80 °C. 

Megamix beads (Catalog No 7801, BioCytex, Marseille, France) measuring 0.5 μm, 0.9 μm, and 3.0 μm and a flow cytometry sub-micron particle size reference kit (Catalog No F13839, Thermo Fisher Scientific, Waltham, MA, USA) for sizes of 0.02 μm, 0.1 μm, 0.2 μm, 0.5 μm, 1.0 μm, and 2.0 μm were used to standardize the setup of the MV analysis region. To distinguish apoptotic MVs from non-apoptotic MVs, plasma was stained using AnnexinV (PerCP-CyTM5.5 Annexin V, Catalog No 561431, BD, San Jose, CA, USA). The microparticles were further stained with the following monoclonal antibodies: (a) vascular endothelial (VE) cadherin (CD144)–PE (clone 11D4.1, Catalog No 561714, BD, San Jose, CA, USA); (b) MUC1 (CD227)–FITC (clone HMPV, Catalog No 559774, BD, San Jose, CA, USA); (c) Tissue Factor (CD142)-APC (clone HTF-1 Catalog No 17-1429-42, eBioscienceTM) and (d) integrin-a2b (CD41a)–PE-Cy™7 (clone HIP8, Catalog No 561424, BD, San Jose, CA, USA). MVs positive for VE-cadherin were identified as endothelial cell-derived microvesicles (EMVs), and MVs positive for integrin-a2b were identified as platelet-derived microvesicles (PMVs). Briefly, the staining protocol was as follows: 10 μL of the patient’s plasma was re-suspended in 190 μL of diluted Annexin V binding buffer 10× concentrate (Catalog No 556454, BD), which originally contained 25 mM of a CaCL2 solution. Then, 5 μL of PerCP-CyTM5.5 -AnnV, 5 μL of (VE)-cadherin (CD144)–PE, 20 μL of MUC1 (CD227)–FITC, 5 μL of Tissue Factor (CD142)-APC, and 10 μL of integrin-a2b (CD41a)–PE-Cy™7 were added. The samples were incubated for 15 min in a dark room at room temperature, and the reaction was stopped by the addition of 400 μL of the diluted binding buffer. The samples were analyzed immediately using a FACSCanto II cytometer (BD Biosciences, San Jose, CA, USA). Data were analyzed from 100,000 events with the aid of FACSDivaTM software, version 6.1.3 (BD). TruCount beads (BDs) were used to calculate the absolute number of circulating MVs in the plasma (Catalog No 340334, BD Pharmingen, San Jose, CA, USA). The specificity of monoclonal antibodies was verified by using identical concentrations of an isotype-matched control antibody to adjust the instrument’s settings, set the fluorescence compensation, and check for instrument sensitivity. 

### 2.5. Statistical Analysis

A statistical analysis was performed using SAS 9.4 for Windows (SAS Institute Inc., Cary, NC, USA). Comparisons of measurements between the various “groups” (patients vs. controls) were performed using the Kruskal–Wallis test to assess differences in measured medical quantities expressed in a numeric form, and to compare proportions when the quantities were expressed in a qualitative manner (i.e., for categorical data), the chi-square test was used. Additionally, wherever dichotomous categorical variables were available, odds ratios were evaluated via Wald’s *p*-value. For correlations, the Spearman correlation coefficient was used as non-parametric tests were preferred in this study. Finally, Kaplan–Meier survival curves were produced, and the log-rank test was used to examine the roles of individual characteristics (i.e., vesicle counts and patient age). The statistical significance level for this study was set at a *p*-value of 0.05.

## 3. Results

### 3.1. Baseline Characteristics

In total, 113 cases were available for this study: 98 patients and 15 controls. Demographic data, medical history data, and routine blood test values for patients and controls are presented in Supplementary Table S1. Despite the fact that the patients had more comorbidities than the controls (2 vs. 1.3, respectively, *p* = 0.046), there were no other significant differences in the above parameters. Therefore, the two populations can be considered matched. The patient group median age was 71 years (IQR 60–79 years) and included 62 male patients (63.3%). The median BMI was 26.6 (IQR 25–28.9), and 39 patients (47.6%) were active smokers. The most common comorbidities were hypertension (52%), heart disease (24.5%), and thyroid disease (17.3%). Blood test results revealed a median hemoglobin value of 11.9 (IQR 10.2–13.7).

### 3.2. Patients and Tumor Characteristics

In patients with CRC, the tumor was located in the left colon, rectum, or right colon in 30.3%, 31.4% and 37.2% of cases, respectively. One case had synchronous colon cancer in the left and right colon. All ninety-eight cases were adenocarcinomas, seven of which were characterized as mucinous adenocarcinomas according to the WHO classification [26]. The majority of the histology reports showed T3 tumors (59.1%), with 18.2% having a T2 tumor, while 5 patients (5.7%) had T in situ (Tis). Lymph node involvement was observed in 44% of patients, with 31.9% categorized as N1 and 12.1% categorized as N2. Tumor cells had intermediate differentiation in 70.9% of patients and high in 23.3% (Table 1). According to the TNM staging system, most patients were staged as stage 2 or 3 (18.0% and 59.0%, respectively), while a rather small percentage (4.0%) had metastases (stage 4). At the last follow up, 70/98 (80.2%) of patients were alive.

### 3.3. Number of Microvesicles in Patients and Controls

**Figure 1:**
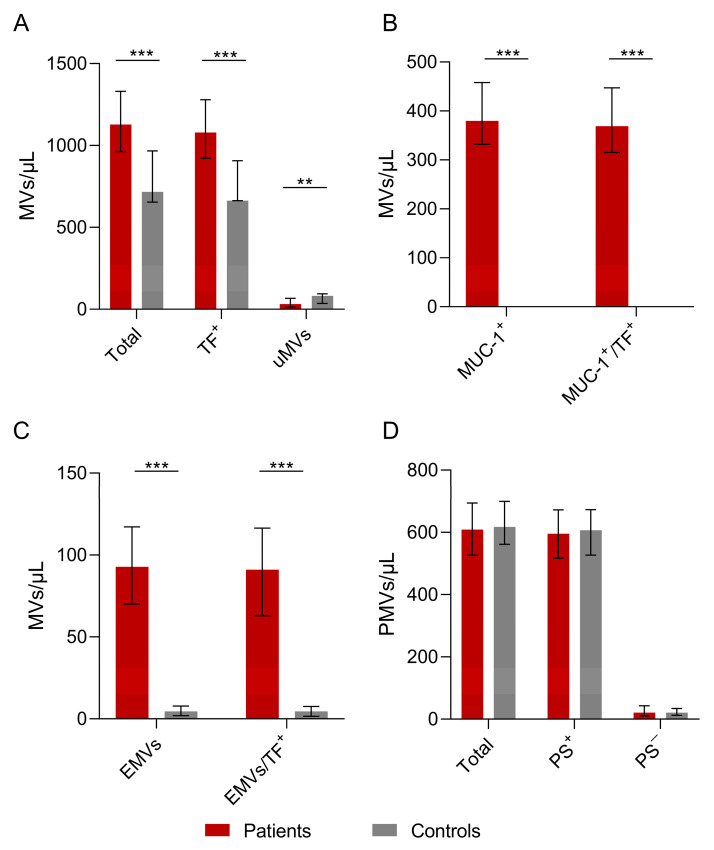
The number of microvesicles in patients’ plasma versus controls. (**A**) The total number of patients’ MVs vs. controls. The numbers of total MVs and TF-positive MVs were higher in patients vs. controls (*p* = 0.000025 vs. *p* = 0.000068). The number of unknown microvesicles (uMVs) was higher in controls than in the patient group (*p* = 0.008). (**B**) Numbers of MUC-1-positive and MUC-1/TF combined MVs were increased in the patients compared to the controls (*p* = 0.000001). (**C**) Statistically significant differences in EMVs and TF-positive EMVs between patients and controls (**D**) Total PMVs, PS^+^ PMVs, and PS^−^ PMVs were not significantly different between patients and controls. Values are shown are medians (bar plots), and error bars represent ± interquartile ranges. *** *p* < 0.001; ** *p* < 0.01.

The MV counts are presented in Table 2 and Figure 1A–D. The total number of MVs was found to be statistically significantly higher in the patients than in the controls. In addition, TF-positive MVs, MUC-1-positive MVs, and EMV counts were also found to be higher in the patients compared to the controls (all *p* < 0.001). The patients’ MUC-1-positive MVs and EMVs co-expressed TF (all *p* < 0.001). Notably, the counts of PMVs and PS-positive or -negative PMVs did not differ significantly between patients and controls. MVs that could not be characterized by the antibodies used in this study, termed unknown microvesicles (uMVs), were lower in patients compared to controls (*p* = 0.008).

### 3.4. Microvesicles in Tis-T1-T2 Patients

**Figure 2:**
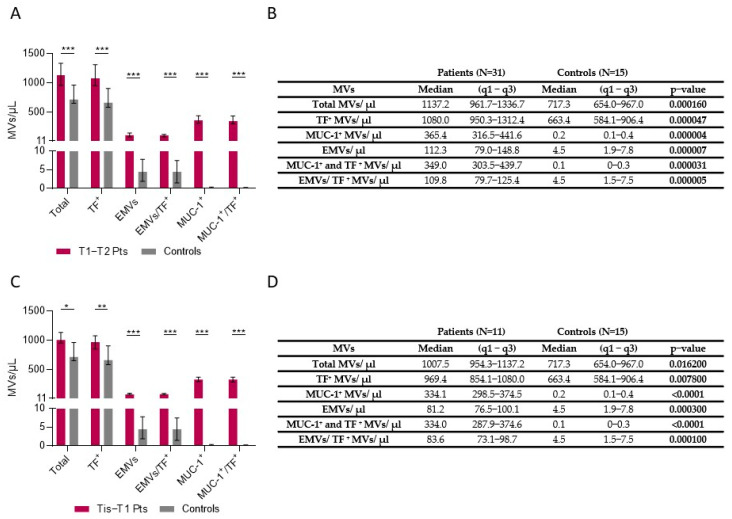
Number of MVs in plasma of Tis-T1-T2 patients vs. controls. (**A**) Patients with early-stage CRC (T1-T2) had higher numbers of total MVs (*p* = 0.00016), Tissue Factor (TF)-positive MVs (*p* = 0.000047), endothelium-positive (*p* = 0.000007) MVs (EMVs), combined EMV/TF MVs (*p* = 0.000005), MUC-1 positive MVs (*p* = 0.000004), and MUC-1/TF combined MVs (*p* = 0.000031) vs. controls. (**B**) MV counts in patients with stages T1 and T2 vs. controls. (**C**) Patients with a very early stage (Tis) of colon cancer (Tis-T1) also had higher numbers of total MVs, TF-positive MVs, EMVs, combined EMV/TF MVs, MUC-1-positive MVs, and combined MUC-1/TF MVs compared to controls (all *p* < 0.05). (**D**) MVs in patients with Tis and stage T1 compared to controls. Values shown in graphs are medians (bar plots), and error bars represent ± interquartile ranges. *** *p* < 0.001, ** *p* < 0.01, and * *p* < 0.05.

Patients with early-stage colon cancer (T1-T2) had statistically significant higher numbers of total MVs, TF-positive MVs, EMVs, combined EMV/TF MVs, MUC-1-positive MVs, and combined MUC-1/TF MVs (Figure 2A,B). Patients with a very early stage of colon cancer (Tis-T1) had also a statistically significant difference regarding total MV counts, TF-positive MVs, EMVs, combined EMV/TF MVs, MUC-1-positive MVs, and MUC-1/TF combined MVs (all *p* < 0.001) (Figure 2C,D).

### 3.5. Microvesicles, Cancer Differentiation, and Lymph Node Invasion

**Figure 3:**
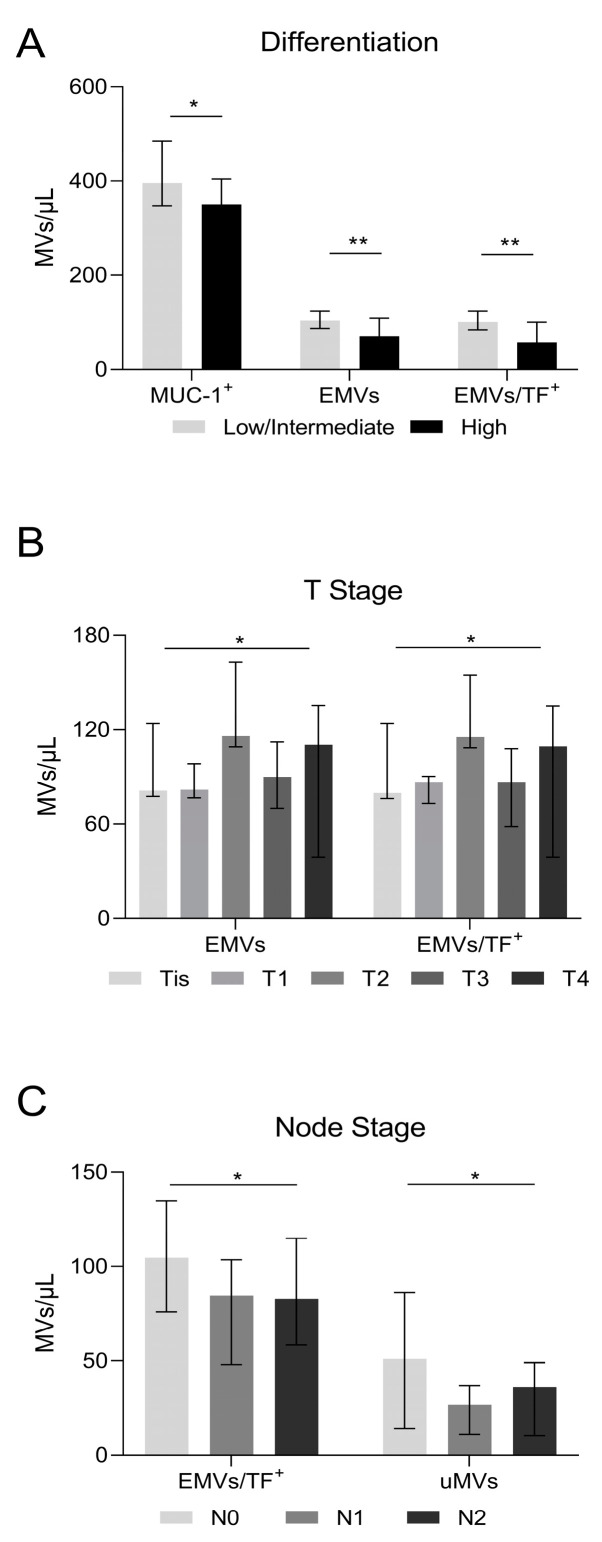
Microvesicle number in relation to cancer differentiation and lymph node invasion. (**A**) Highly differentiated tumors had lower MUC-1-positive MVs (350/μL vs. 418/μL in low/intermediate, *p* = 0.027); endothelial cell-derived microvesicles (EMVs) (70/μL vs. 115/μL in low/intermediate, *p* = 0.0027), and combined EMV/TF MVs (57/μL vs. 110/μL in low/intermediate, *p* = 0.0018). (**B**) MVs in relation to the tumor size. EMVs detected either alone or in combination with TF-positive MVs showed differences (Tis patients: EMVs = 81.2/μL and EMV/TF MVs = 79.7/μL; T1 patients: EMVs = 81.8/μL and EMV/TF MVs = 86.4/μL; T2 patients: EMVs = 116/μL and EMV/TF MVs = 115.4/μL; T3 patients: EMVs = 90.1/μL and EMV/TF MVs = 86.4/μL; T4 patients: EMVs = 110.4/μL and EMV/TF MVs = 109.4/μL; *p* = 0.04 and *p* = 0.02, respectively). (**C**) Patients with lymph node invasion had lower counts of EMV/TF combined positive MVs and uMVs. (Node = 0: EMV/TF MVs = 104.7/μL and uMVs 51.1/μL; Node = 1: EMV/TF MVs = 84.7/μL and uMVs 26.8/μL; Node = 2: EMV/TF MVs = 82.9/μL and uMVs 36.2/μL; *p* = 0.03 and *p* = 0.04, respectively). Values are medians (bar plots), and error bars represent a ± interquartile range. ** *p* < 0.01, and * *p* < 0.05.

As only 5.8% of patients had a low level of tumor differentiation (Table 1), we compared cases with high differentiation with those with intermediate or low levels of differentiation (combined). According to our results, patients with highly differentiated tumors had lower counts of MUC-1-positive MVs (*p* = 0.027), EMVs (*p* = 0.0027), and combined EMV/TF MVs (*p* = 0.0018) compared to those with low/intermediate differentiated tumors (Figure 3A). MV levels in relation to tumor size Tis, T1-4 are shown in Figure 3B. Patients with tumor lymph node invasion had lower counts of EMV/TF MVs bearing MVs as well as uMVs (Figure 3C).

### 3.6. Microvesicles and Patient Survival

**Figure 4:**
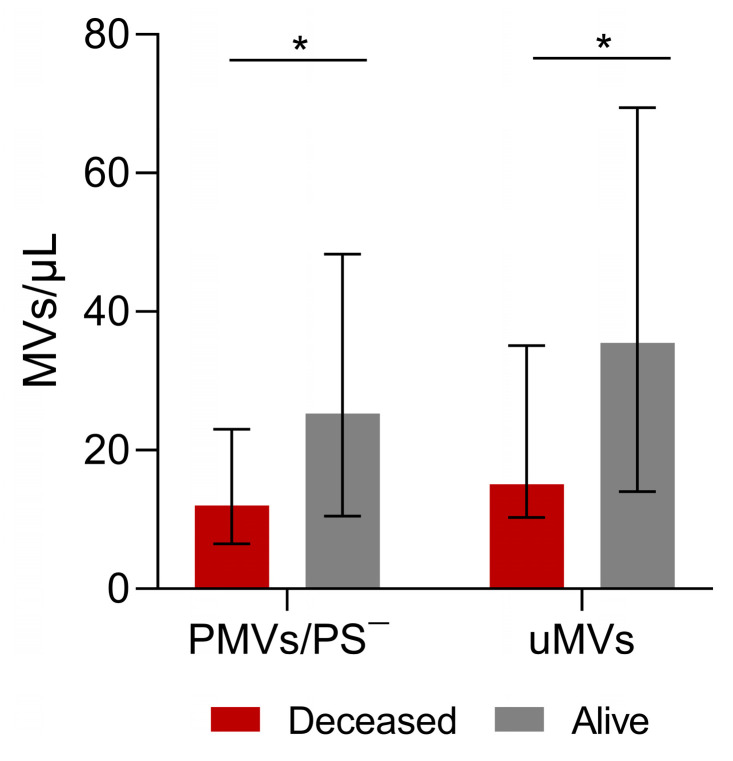
Analysis of microvesicles and blood test measurements in surviving and deceased patients. Phosphatidylserine (PS)-negative platelet MVs (PMVs) and unknown MVs in patients who survived and patients who died. Patients who remained alive had more PS-negative PMVs and more uMVs. PMVs: Alive—25.2/μL; Deceased—12.0/μL; *p* = 0.03. unMVs: Alive—35.5/μL; Deceased—15.1/μL; *p* = 0.02). Values are medians (bar plots), and error bars represent ± interquartile ranges. * *p* < 0.05.

Regarding patient survival, 19 patients died, with a mean time to death of 4.5 years (SE: 0.1 years) and with no difference between men and women (similar survival distribution functions)(log-rank test *p* = 0.9669; for men: mean time to death—4.6 years and SE—0.2 years; for women: mean time to death—3.7 years and SE—0.2 years). The univariate log-rank test for the role of each type of MV identified and assessed in our study as well as patient age showed that none of these variables had a significant role in patient survival. Comparing data between the 19 patients who died and those who survived, PS-negative PMV and uMV levels were lower in patients who died (*p* = 0.0317 and *p* = 0.0254 respectively, Figure 4). 

## 4. Discussion

In cancer patients, microvesicles arise from multiple cellular sources within the tumor microenvironment and play crucial roles in cancer pathogenesis and progression [27,28]. Microvesicles in cancer patients can originate from both tumor cells and non-tumor cells [29,30,31]. Tumor-derived microvesicles are directly released by cancer cells and carry specific molecular signatures reflective of the tumor’s genetic and phenotypic characteristics [29,32,33,34]. On the other hand, microvesicles released by non-tumor cells in response to the tumor microenvironment represent the body’s reaction to the tumor [32,33,35,36].

In this prospective study, we identified and assessed MV levels in the blood of CRC patients at diagnosis and compared the results with a control group. We also investigated the possible association of MVs with survival in these patients. The two populations were matched, as stated in the results section, to control for an important confounding factor, which was age-related decline in MV concentration [37].

Our study demonstrated that prior to any treatment, the CRC patients had higher plasma levels of total MVs, TF-positive MVs, MUC-1-positive MVs, and EMVs than the controls (*p* < 0.001). PMV levels were not significantly different between the two groups (patients vs. controls). Our results are also supported by findings from Zhao et al. [38], who showed significantly higher plasma levels of PS-positive MVs and EMVs in colon cancer patients at all stages compared to a control group. They also found statistically significantly higher levels of PS-positive PMVs in colon cancer patients, which is not supported by our data. This may be due to the different methods used to isolate PS-rich cells and MVs. In our study, we used Annexin V, whereas Zhao et al. [38] used lactadherin, which has been shown to be more effective in isolating MVs [39,40]. Eddama et al., 2022, similarly demonstrated elevated levels of MVs in CRC patients, supporting our data and further pointing out the role of MVs as a potential biomarker for early disease detection [21].

According to our study, patients with early-stage colorectal cancer (T1-T2) as well as patients with very early-stage colorectal cancer (Tis-T1) had higher total numbers of MVs, EMVs, MUC-1-positive MVs, TF-positive MVs, EMV/TF combined MVs, and MUC-1/TF combined MVs compared to controls. To the best of our knowledge, this is the first study to show statistically significant differences in MV levels in patients at a very early stage of colorectal cancer versus controls. This novel finding could indicate a possible diagnostic biomarker that may allow for the early detection of tumors.

MVs released by tumor cells (TMVs) may be of paramount importance for diagnostic and therapeutic modalities but are estimated to represent only 1% of the host’s MVs [7]. MUC-1 is a tumor marker overexpressed in colon cancer [41,42], and our data show that MUC-1 positive MVs are statistically significantly increased in CRC patients, even in the early stages of the disease. For these reasons, MUC-1 may be a promising early biomarker or therapeutic target [43,44,45]. This has also been suggested by other studies. Stec et al. [46] found that MVs from CRC patients expressed tumor markers such as HER-2/neu, MUC-1, and EGFR at much higher levels than those from healthy individuals. In addition, EGFR expression was detected by Western blotting and not by flow cytometry, probably because it is not expressed on the surface of the MV [46]. 

TF-positive MVs, which are associated with an increased risk of thrombosis in cancer patients due to their procoagulant properties [47], were found at significantly higher levels in CRC patients in our study as well as others [38,48,49,50]. Hisada et al. [50] also found that patients with adenocarcinoma had statistically significantly higher levels of TF-positive MVs than subjects with other cancer types, possibly due to high levels of expression of TF on the surfaces of epithelial cells. They also showed that cancer patients with high levels of TF MV activity had better prognoses than those with low levels (*p* < 0.0433) [50].

Our results show that highly differentiated tumors had fewer MUC-1-positive MVs, EMVs, and combined EMV/TF microvesicles than ones with intermediate/low differentiation. Furthermore, our results support the role of PS-negative PMVs (*p* = 0.0317) and the small count of uMVs in patient survival (*p* = 0.0254) (Figure 4). The significance of PS-negative MVs is still unclear; however, it is possible that they also play other roles beyond procoagulant phospholipid activity [51]. The level of circulating MVs could be used as a tumor indicator as it correlates with poor prognosis parameters and shorter survival [52]. Helley et al. [53] found that baseline levels of PMVs were significantly associated with survival in prostate cancer patients.

EMVs have been found to be elevated in metastatic colorectal cancer patients, and Nanou et al. [54] even highlighted their role as predictors of overall survival that could contribute to decision making. They are released in stages II, III, and IV of CRC during VEGF-stimulation mediated endothelial proliferation and tumor proliferation [39,55,56]. In this context, Zhao et al. [38] found increased levels of EMVs in colon cancer patients with stage II, III, or IV cancer but not in stage I. However, this finding could not be confirmed in our study due to the small number of metastatic colon cancer patients included.

The study was constrained by a relatively small number of cases, which may restrict the generalizability of our findings to broader populations. The relatively small sizes of the two groups, however well defined (patients and controls), were primarily due to logistical challenges in data recruitment and collection. Accordingly, our study predominantly focused on cases with familiar histology and specific molecular profiles, potentially limiting the applicability of our findings to other histologic subtypes or molecular subgroups of the disease. 

With continued improvements in isolation techniques, biomarker discovery, and validation studies, microvesicles hold significant promise as possible non-invasive biomarkers for cancer diagnosis and prognosis in the future [30,57,58]. Overall, resolving these issues and addressing associated issues requires interdisciplinary collaboration, rigorous validation studies, and concerted efforts to standardize methodologies and promote transparency in reporting [59,60]. By overcoming these challenges, the field of MV research could realize its full potential in advancing precision medicine and improving cancer diagnosis, prognosis, and treatment [61,62,63,64]. 

## 5. Conclusions

In conclusion, our study reveals that CRC patients present at diagnosis with elevated levels of MVs, namely total MVs, MUC-1-positive MVs, TF-positive MVs, and EMVs, with statistical significance compared to controls, even in very early stages of the disease. As the identification of tumor indicators to detect the presence of disease using noninvasive diagnostic procedures plays a pivotal role in cancer research, our findings suggest that analyzing circulating MV levels in plasma could serve as a valuable tool for the early diagnosis of colorectal cancer, particularly in the initial stage of the disease. However, standardizing isolation methods, and the establishment of robust biomarker panels along with additional studies encompassing a broader spectrum of histologic and molecular phenotypes are warranted to comprehensively confirm our findings and the clinical relevance of microvesicle profiles in CRC patients.

## Figures and Tables

**Table 1:** Collective presentation of patient description and tumor characteristics (%). The tumor differentiation was characterized as low, intermediate and high. The TNM classification was used for staging of the cancer.

Disease Related Characteristics of Patients (%)									
Tumor Differentiation									
Low				Intermediate			High		
5.8				70.9			23.3		
Tumor location									
Left C			Rectal			Right C		Left and right C	
30.3			31.4			37.2		1.1	
Tumor growth									
T1-T2					T3-T4				
34.1					65.9				
TNM staging system									
Tis		T1		Τ2		T3		T4	
5.7		6.8		18.2		59.1		10.2	
Lymph node invasion									
Negative					Positive				
56.0					44.0				
TNM staging system: node									
N0				N1			N2		
56.0				31.9			12.1		
TNM staging system: metastasis									
Yes					No				
3.9					96.1				
Patient alive during last follow up									
80.2									

**Table 2:** Number of microvesicles in patients’ plasma and in controls. Numbers of MVs are presented as median values with (q1 and q3 quartiles). Statistically significant values are shown in bold.

	Patients (N = 98)		Controls (N = 15)		
MVs	Median	(q1–q3)	Median	(q1–q3)	*p*-Value
Total MVs/μL	1127.7	962.9–1331.2	717.3	654–967	**0.000068**
TF^+^ MVs/μL	1078.8	922.4–1278.8	663.4	584.1–906.4	**0.000025**
MUC-1^+^ MVs/μL	379.8	332–458.3	0.2	0.1–0.4	**0.000000**
EMVs/μL	92.8	70.1–117.2	4.5	1.9–7.8	**0.000001**
MUC-1^+^ and TF^+^ MVs/μL	368.8	315.2–447.4	0.1	0–0.3	**0.000000**
EMVs/TF^+^ MVs/μL	91.1	62.9–116.4	4.5	1.5–7.5	**0.000001**
uMVs/μL	31.8	13.3–66.6	81.4	35.1–94.1	**0.008592**
PMVs PS^+^/μL	595.4	517.3–672.5	606.7	527.2–673	0.475837
PMVs PS^−^/μL	21.1	10–42.9	21.0	12.1–34.5	0.971382
PMVs/μL	608.7	526.7–694.5	617.4	561.6–699.6	0.384319

## Data Availability

The data presented in this study are available upon reasonable request.

## Supplementary Materials

The following supporting information can be downloaded at: https://www.mdpi.com/article/10.3390/cancers16101943/s1, Table S1: Demographic data, medical history data and blood test information, for patients and controls. Numerical data are presented as median (q1 and q3 quartiles), and categorical data as corresponding percentages. *p* values, are presented (*). Statistically significant values are shown in bold.
