# Supplementary material for: Precision Oncology: Circulating Microvesicles as New Biomarkers in a Very Early Stage of Colorectal Cancer
Source: Cancers (Basel). 2024 May 20;16(10):1943. doi: 10.3390/cancers16101943 (PMC11119677; doi:10.3390/cancers16101943)
Supplement: Supplementary file 1 [file cancers-16-01943-s001.zip › cancers-2998738-supplementary.pdf]

Supplementary **Table S1.** Demographic data, medical history data and blood test information, for patients and controls. Numerical data are presented as median (q1 and q3 quartiles), and categorical data as corresponding percentages. *p* values, are presented (*). Statistically significant values are shown in bold.

		Patients (N=98)		Controls (N=15)		p-value*
**Demographics**	Gender male	63.3 %		66.7 %		0.7986
	Smoking %	47.0 %		40.0 %		0.7454
		**Patients (N=98)**		**Controls (N=15)**		
	**Characteristic**	**Median**	**(q1 - q3)**	**Median**	**(q1 - q3)**	**p-value**
	Patient age (years)	71.0	60.0-79.0	68.0	56.0-74.0	0.192298
	Weight (Kgr)	79.5	70.0-86.0	77.0	70.0-80.0	0.389757
	Height (cm)	165.0	160.0-175.0	167.5	160.0-175.0	0.977044
	Body Mass Index (Kgr/m^2)	26.6	25.0-28.9	26.6	25.1-28.0	0.709609
**Blood test results**	White Blood Cells (K/μl)	7.0	5.5-8.2	5.8	4.8-7.9	0.274150
	Neutrophils (%)	61.0	55.0-67.6	61.0	58.0-70.0	0.608098
	Lymphocytes (%)	28.0	20.0-34.4	27.0	22.2-29.7	0.521675
	Monocytes (%)	8.0	5.5-9.4	8.0	5.6-9	0.908180
	Eosinophils (%)	2.0	1.0-3.1	3.0	1.3-4.7	0.507900
	Basophils (%)	1.0	0.6-1.3	1.0	0.6-0.9	0.062515
	Hemoglobin (g/dL)	11.9	10.2-13.7	13.3	12.0-14	0.121943
	Hematocrit (%)	37.2	31.9-42.6	40.9	36.3-42.1	0.228832
	Platelets (K/μl)	257.0	209.0-325	242.0	196-284.0	0.142857
	Fibrinogen (mg/dl)	357.1	296.9-436.7	346.6	302.0-402	0.492650
	D-dimers (μg/l)	380.0	260.0-710.0	370.0	200.0-590	0.384857
**Medical record**		**N**	**%**	**N**	**%**	
	Hypertension	51	52.0%	5	33.3	0.2675
	Prostate	11	11.2%	0	0.0	0.354400
	Thyroid disease	17	17.3%	2	13.3	> 0.999
	Dyslipidaimia	18	18.4%	1	6.7	0.4598
	Diabetes	16	16.3%	2	13.3	> 0.999
	Cancer history	6	6.1%	0	0.0	> 0.999
	Heart Disease	24	24.5%	3	20.0	> 0.999
	Abnormal cholesterol	9	9.2%	1	6.7	> 0.999
	Other diseases	46	46.9%	4	26.7%	0.1707
		**Median**	**Range**	**Median**	**Range**	
	Number of comorbidities	2.0	1.0-3.0	1.0	0.0-3.0	**0.046155**
